# Discriminant Validity of the Standardized On-Road Assessment for Drivers (SOAD) Among Stroke Patients in Japan

**DOI:** 10.7759/cureus.75170

**Published:** 2024-12-05

**Authors:** Tatsunori Sawada, Kana Sakaue, Junpei Kondou, Yuki Higashikawa, Kanta Ohno, Kounosuke Tomori

**Affiliations:** 1 Department of Rehabilitation, Tokyo University of Technology, Tokyo, JPN; 2 Department of Rehabilitation, Shonan Keiiku Hospital, Fujisawa, JPN; 3 Department of Rehabilitation and Care, Hatsudai Rehabilitation Hospital, Tokyo, JPN; 4 Department of Rehabilitation, Nakaizu Rehabilitation Center, Izu, JPN

**Keywords:** closed-course, cognitive function, discriminant validity, driving assessment, occupational therapy, on-road test, rehabilitation, soad, stroke

## Abstract

Introduction: On-road tests are considered the gold standard for evaluating real-world driving skills. However, their reliability and validity remain inadequately established, particularly under varying legal and road conditions across countries.

Aim: This study investigates the discriminant validity of the closed-course version of the Standardized On-Road Assessment for Drivers (SOAD) in Japan.

Methods: This study was conducted in five Japanese rehabilitation hospitals and affiliated driving schools. The participants consisted of 108 brain-injured individuals (mean age: 50.0 years) undergoing driving assessments. The inclusion criteria focused on physician-referred patients diagnosed with brain injuries. The SOAD closed-course test, consisting of 40 items, was compared with off-road cognitive assessments, including the Mini-Mental State Examination Japanese Version (MMSE-J), Trail Making Test Japanese Version (TMT-J), Rey-Osterrieth Complex Figure Test, Stroke Drivers Screening Assessment Japanese version (J-SDSA), and Kohs Block Design Test. Spearman's correlation was used to evaluate discriminant validity, distinguishing driving-specific skills from cognitive functions.

Results: Weak to moderate correlations were found between SOAD and off-road tests, supporting the discriminant validity of SOAD. Among off-road tests, the J-SDSA dot time correlated most frequently with SOAD items, followed by MMSE-J and TMT-J. The highest correlation coefficient (-0.38) was observed between the J-SDSA dot error and a specific SOAD item.

Conclusion: These results show that SOAD demonstrates strong discriminant validity as a closed-course on-road assessment tool for brain-injured individuals and measures unique aspects of driving skills not captured by cognitive tests.

## Introduction

In numerous countries, individuals with brain injuries are a primary focus of rehabilitation, and driving assessments play an important role in occupational therapy. Post-stroke evaluations encompass off-road tests, such as neuropsychological examinations and driving simulators, and on-road tests involving actual driving alongside physical function assessments [1-3]. The on-road test is considered the gold standard for examining real-world driving skills among these assessments [4,5]. Because various studies have aimed to determine the predictability of on-road test results [5-8], it is an essential factor to ensure the reliability and validity of on-road tests.

While some on-road tests for brain-injured individuals have been developed globally, only a few have been validated with a high degree of reliability and validity. A systematic review identified a scarcity of on-road tests demonstrating general reliability and validity [9]. This scarcity suggests that a few on-road tests may partially exhibit reliability and validity. A subsequent study by Bellagamba et al. [10] reinforced these findings, concluding that currently available on-road tests lack sufficient reliability and validity. Consequently, the reliability and validity of on-road tests remain inadequately established.

Despite uncertainties regarding the reliability and validity of the on-road tests themselves, past efforts have focused on predicting driving skill assessment outcomes using off-road tests. Studies have attempted to formulate prediction equations using multiple off-road tests to predict actual on-road evaluation outcomes [5-8]. However, this methodology cannot inherently verify the validity of the on-road test, leaving the authenticity of the results unverified. Consequently, these results are utilized as predictive tools without confirming sufficient validity. Alternatively, exploring alternative conceptual frameworks that involve both on-road and off-road tests introduces the crucial aspect of discriminant validity. Discriminant validity, a statistical concept, assesses whether a measurement captures unique attributes distinct from other related measurements [11]. Several previous studies have used discriminant validity to demonstrate the validity of driving evaluations, supporting the validity of these assessments [7,12,13]. Instead of relying on outcome predictions derived from past on-road test results as observed in previous studies, it is more beneficial to authenticate whether the on-road test effectively encapsulates the essence of driving skills, embodying discriminant validity. This methodology enhances the assurance of on-road test validity.

Taking an alternative perspective, the legal and road conditions in different countries contribute to the complexity of on-road testing. On-road tests evaluate several key components of driving, such as cognitive abilities (e.g., attention, decision-making, and visual perception), motor skills (e.g., pedal and steering control), and environmental awareness (e.g., responding to vehicles and pedestrians, adherence to traffic rules) [14-16]. Eligibility criteria for these assessments often include a confirmed diagnosis of brain injury, sufficient physical ability to operate a vehicle, and adequate cognitive functioning to manage driving-related tasks. However, the specific criteria for determining eligibility for driving assessments vary across countries or institutes, reflecting differences in legal regulations, cultural contexts, and healthcare systems. Existing on-road tests are primarily conducted on actual roads, exposing brain-injured patients to various hazards such as other vehicles and road users. In contrast, Japan conducts some on-road tests in a closed course at driving schools under instructor guidance. Legal regulations in Japan may restrict brain-injured survivors from driving on public roads until they pass an aptitude test from the local public safety commission [17]. These legal constraints necessitate the assessment of on-road tests for brain-injured individuals solely on closed courses within driving schools [18]. In other situations, other countries have difficulty conducting on-road tests under actual road conditions due to legal regulations [19].

Considering scenarios where specific countries are restricted from public on-road tests, a selective and comprehensive assessment approach incorporating both closed-course (driving school) and public-course on-road tests becomes preferable. However, most existing on-road tests have omitted closed courses, and when included, they often involve simple driving in a parking lot [20,21]. While public on-road tests in real driving settings are valuable, regulatory frameworks in some countries may limit their feasibility. In such cases, on-road tests within a driving school setting offer a valuable alternative to assess critical driving skills, including operational proficiency and attentiveness.

In response to these problems, we developed the Standardized On-Road Assessment for Drivers (SOAD), incorporating both closed-course and public-course on-road tests, and demonstrated its content validity [22]. Given that no on-road tests with established reliability and validity exist, the investigation into the discriminant validity of SOAD becomes significant. The presence of a valid on-road test for a closed course could prove advantageous, especially in countries facing legal constraints such as Japan. Therefore, the primary objective of this study is to scrutinize the discriminant validity of the closed course version, contributing to the further validation of SOAD.

## Materials and methods

Design and setting

This study used a cross-sectional design. We conducted this study at five rehabilitation hospitals and their affiliated driving schools in Japan. This study was approved by the Ethics Committee of the Tokyo University of Technology (No. E21HS-019), and written informed consent was obtained from all participants in the study.

Object

The inclusion criteria were as follows. 1) Patients referred by a physician for driving assessment at the collaborating hospital, 2) patients who were primarily diagnosed with a stroke, and 3) patients who will undergo an on-road assessment. The exclusion criteria were 1) patients with severe cognitive impairments that prevent understanding of the research content, 2) patients with aphasia that would interfere with the assessments, and 3) patients who do not provide consent for participation in this study. Although vision is a factor that influences driving, it was not included as a criterion in the present study. Instead, the focus was placed on examining correlations with the off-road tests utilized in previous driving studies.

SOAD

SOAD was developed through the Delphi method, incorporating items from previous on-road assessments and insights gathered from experts. The content validity of SOAD has been substantiated through this process [22]. The SOAD on the closed course (in driving school) involves scoring a total of 40 items, comprising eight categories related to driving (physical function, cognitive functions, mechanical operation, driving attitude, vehicle position, following the traffic rule, basic driving, safety confirmation) and four items related to dangerous action (Table 1). SOAD items cover detailed aspects of driving performance, such as operational proficiency, attentiveness, and adherence to traffic rules. Their comprehensive descriptions are provided in the SOAD manual.

**Table 1:** SOAD closed course items SOAD: Standardized On-Road Assessment for Drivers

No.	Category	Content
1	Physical function	Have the durability to drive
2		Maintain appropriate posture for safe driving
3	Cognitive function	No attention problems
4		Judge safety
5		Solve problems
6		Remember route/instruction(memory)
7	Mechanical operation	Operate the handle appropriately
8		Operate indicators appropriately
9		Operate wipers appropriately*
10		Operate brakes appropriately
11		Operate accelerators appropriately
12		Operate a clutch appropriately*
13		Operate a gear change appropriately
14		Operate an assistive device installed appropriately*
15	Driving attitude	Being aware of appropriate attitude to drive safety
16		Having sufficient confidence to drive safely
17		Predict hazard
18		Follow instructions
19		Being aware of mistakes made
20	Vehicle position	Position in the lane appropriately
21		Select the correct lane appropriately
22		Maintain appropriate distance
23		Pass over the marker appropriately*
24	Follow the traffic rule	Follow traffic signal appropriately
25		Follow traffic sign/road marking appropriately
26		Follow stop sign appropriately
27		Follow yield situation appropriately
28		Slow down appropriately
29	Basic driving	Start a car appropriately
30		Reverse park appropriately
31		Adjust driving speed appropriately
32		Sudden brake appropriately
33		Drive pass the narrow street appropriately
34	Safety confirmation	Check rear view
35		Check safety using head/body movement
36		Check safety when turing right
37		Check safety when turing left
38		Check safety when changing lane/merging
39		Check safety when starting to drive
40		Check safety in poor visibility
41	Dangerous action	No instances of driving the wrong way
42		No accidental pressing of the accelerator and brake
43		No near-misses of accidents (property damage or personal injury)
44		No instances of running red lights
*Situation-dependent optional item, dangerous actions are scored on a binary scale.		

SOAD assessments were conducted using a training vehicle equipped with auxiliary brakes. SOAD on the closed course utilizes the course outlined in the provisional license test as specified by Japanese regulations, which includes maneuvers such as changing the vehicle's direction and navigating through narrow passages. Participants drive the course twice, receiving brief feedback from the assessor after the first attempt and then proceeding to the second attempt. Scoring is based on whether there were mistakes on the items during the first and second attempts. Items are scored on a three-point scale (zero to two points), while dangerous actions are scored on a binary scale (presence or absence). A high score means good driving skills.

Data collection

In this study, several off-road tests were adopted as alternative conceptual frameworks for discriminant validity. The off-road tests utilized in this study are the Mini-Mental State Examination Japanese Version (MMSE-J), Trail Making Test Japanese Version (TMT-J), Kohs Block Design Test (KBDT), Rey-Osterrieth Complex Figure Test (ROCF), Frontal Assessment Battery (FAB), and Stroke Drivers’ Screening Assessment Japanese Version (J-SDSA), which have been commonly employed in previous research for guessing driving skills in individuals with brain injuries [1,2,5]. However, these assessments are conducted to evaluate the cognitive functions (higher brain functions) of stroke patients and do not measure the actual driving skills. Since on-road tests are often compared with evaluations by driving instructors, their correlation with off-road tests can help establish discriminant validity.

For the on-road assessment using SOAD, driving courses at authorized Japanese driving schools affiliated with each hospital were utilized. The assessment was conducted with a vehicle equipped with auxiliary brakes, with the presence of an occupational therapist and a driving instructor. The SOAD assessment was scored by a trained occupational therapist, while the off-road tests were administered by an occupational therapist in the collaborating hospital.

Data analysis

Statistical analyses of the discriminant validity were conducted by the research team (TS, KS, JK, YH) independent of the evaluators. Data were managed in the cloud, and the research team carried out the analysis via online meetings. In this study, the score for each item and each category score from the 40 usual items, as well as the scores from each off-road test, were used as variables. Following confirmation of normality, the correlation coefficient was employed for statistical analysis by the Spearman Ranked test. All statistical analyses were performed with IBM SPSS Statistics for Windows, Version 24 (Released 2016; IBM Corp., Armonk, New York, United States) with a significance level of 5%.

## Results

The study group consisted of 108 stroke patients, 83 men and 25 women, with ages ranging from 34 to 80 years (mean: 50.0 years, SD: 10.2). The participants' diagnoses were as follows: 62 individuals were diagnosed with cerebral infarction, 26 with cerebral hemorrhage, 10 with subarachnoid hemorrhage, three each with traumatic brain injury, encephalitis, and brain tumor, and one individual with hydrocephalus. The average driving experience of the participants was 36.3 (SD: 11.0) years. The means and standard deviations of the off-road test are shown in Table 2. There were two missing values in the FAB, and the optional items of SOAD have some missing values (60 for item 9, 102 for item 12, 87 for item 14, and 26 for item 23).

**Table 2:** Average of off-road test MMSE-J: Mini-Mental State Examination Japanese Version; TMT: Trail Making Test; KBDT: Kohs Block Design Test; ROCF: Rey-Osterrieth Complex Figure Test; FAB: Frontal Assessment Battery; J-SDSA: Stroke Driver's Screening Assessment Japanese Version

Off-road test	Mean	SD
MMSE-J	28.8	1.8
TMT-A	48.2	20.8
TMT-B	86	48.3
KBDT	103	16.6
ROCF copy	34	3.2
ROCF recall	21.1	7.1
FAB	16.6	2.2
J-SDSA dot time	452.4	138.8
J-SDSA dot errors	8.6	11
J-SDSA dot false positives	0.3	0.8
J-SDSA compass	30.5	4.7
J-SDSA square	26.1	5.9
J-SDSA road sign	8.3	2

Several correlations were observed between the items of the SOAD and various off-road tests, though most of these correlations were not statistically significant (Table 3). Among the off-road tests, the J-SDSA dot time showed the highest number of significant correlations with six items (maintain the appropriate posture for safe driving, operate a gear change appropriately, position in the lane appropriately, maintain appropriate distance, slow down appropriately, check safety when turning left), followed by the MMSE-J with four items (operate brakes appropriately, reverse park appropriately, check rearview, check safety using head/body movement), and the TMT-J with three significant items (TMT-A: operate a gear change appropriately, follow yield situation appropriately, check safety using head/body movement, TMT-B: operate indicators appropriately, slow down appropriately, check safety when starting to drive).

**Table 3:** The correlation coefficient (r) between SOAD item and off-road tests MMSE-J: Mini-Mental State Examination Japanese Version; TMT: Trail Making Test; KBDT: Kohs Block Design Test; ROCF: Rey-Osterrieth Complex Figure Test; FAB: Frontal Assessment Battery; J-SDSA: Stroke Driver's Screening Assessment Japanese Version; SOAD: Standardized On-Road Assessment for Drivers *p < 0.05, **p < 0.01 A correlation coefficient (r) is considered weak if r < 0.3, moderate if 0.3 ≦ r < 0.7, and strong if r ≧ 0.7.

No.	Assessment items	MMSE-J	TMT-A	TMT-B	KBDT	ROCF copy	ROCF recall	FAB	J-SDSA dot time	J-SDSA dot errors	J-SDSA dot false positives	J-SDSA compass	J-SDSA square	J-SDSA road sign
Item 1	Have the durability to drive	0.00	-0.05	0.00	0.01	0.02	-0.09	-0.19	-0.05	-0.07	-0.15	-0.02	0.06	-0.01
Item 2	Maintain appropriate posture for safe driving	0.08	-0.04	0.00	0.03	0.05	-0.03	-0.06	-0.25*	0.03	-0.04	0.08	0.02	0.09
Item 3	No attention problems	-0.02	0.05	-0.08	-0.01	-0.01	-0.04	-0.11	-0.14	0.01	0.11	-0.17	-0.08	0.06
Item 4	Judge safety	-0.02	-0.10	-0.05	0.13	-0.01	0.00	-0.09	-0.05	-0.03	0.09	-0.08	-0.15	-0.09
Item 5	Solve problems	-0.08	0.03	-0.03	-0.02	0.03	-0.03	-0.03	-0.06	0.02	0.17	-0.19	-0.09	-0.05
Item 6	Remember route/instruction (memory)	0.01	-0.14	-0.07	0.12	0.07	0.13	-0.11	-0.08	0.00	0.07	-0.04	0.17	0.15
Item 7	Operate the handle appropriately	-0.06	-0.04	-0.07	0.07	-0.05	0.03	-0.08	-0.10	-0.03	-0.20*	-0.10	-0.01	-0.05
Item 8	Operate indicators appropriately	-0.05	-0.10	-0.21*	0.06	0.09	-0.08	0.00	-0.16	-0.12	0.01	0.01	0.15	0.08
Item 9	Operate wipers appropriately	-0.13	-0.12	0.02	0.15	-0.02	0.22	0.00	-0.19	-0.08	0.08	-0.08	0.18	0.01
Item 10	Operate brakes appropriately	-0.29**	0.09	-0.02	-0.06	0.10	-0.08	-0.12	-0.07	-0.05	-0.01	0.08	-0.05	-0.09
Item 11	Operate accelerators appropriately	-0.18	0.13	0.04	-0.16	-0.09	-0.20	-0.15	-0.11	-0.02	0.05	0.13	-0.17	-0.08
Item 12	Operate a clutch appropriately	-0.48	0.41	-0.51	0.14	0.26	0.38	0.51	-0.44	-0.55	-0.46	-0.31	-0.68	-0.42
Item 13	Operate a gear change appropriately	-0.05	0.23*	0.18	-0.16	-0.28**	0.03	-0.16	0.23*	0.05	-0.09	-0.11	-0.10	-0.18
Item 14	Operate an assistive device installed appropriately	-0.14	0.00	-0.28	-0.04	-0.01	0.22	0.12	-0.18	-0.05	-0.18	0.15	0.23	-0.06
Item 15	Being aware of appropriate attitude to drive safety	-0.08	-0.03	0.06	0.03	-0.04	-0.07	-0.09	-0.01	0.00	0.01	0.02	0.11	0.04
Item 16	Having sufficient confidence to drive safely	0.00	0.06	0.09	-0.08	-0.13	-0.19	-0.08	0.04	0.01	-0.02	-0.04	0.00	0.04
Item 17	Predict hazard	-0.07	-0.08	-0.15	0.11	0.10	-0.01	-0.04	-0.13	0.01	0.11	-0.13	-0.01	0.13
Item 18	Follow instructions	0.14	0.03	0.03	-0.02	-0.11	-0.09	-0.01	-0.08	0.09	0.10	0.03	-0.07	-0.12
Item 19	Being aware of mistakes made	-0.01	0.00	-0.05	-0.04	-0.06	0.00	-0.13	-0.02	0.02	0.12	-0.14	0.10	0.08
Item 20	Position in the lane appropriately	-0.12	-0.11	-0.15	0.17	0.19	0.06	0.00	-0.32**	-0.08	0.01	0.03	0.12	0.05
Item 21	Select the correct lane appropriately	-0.04	-0.19	-0.15	-0.02	0.10	0.00	-0.09	-0.17	-0.06	-0.15	-0.14	0.10	-0.03
Item 22	Maintain appropriate distance	-0.07	-0.03	-0.12	-0.05	0.00	0.08	-0.03	-0.20*	-0.04	-0.01	-0.10	0.03	-0.01
Item 23	Pass over the marker appropriately	0.09	0.01	-0.03	-0.01	-0.10	-0.01	-0.05	-0.07	0.01	-0.05	-0.02	0.15	0.16
Item 24	Follow traffic signal appropriately	0.00	0.19	-0.09	0.00	-0.09	-0.02	0.11	0.13	0.09	0.00	-0.19	-0.10	-0.17
Item 25	Follow traffic sign/road marking appropriately	-0.02	0.00	-0.03	0.03	0.02	0.07	-0.11	-0.07	-0.05	-0.11	-0.09	0.04	0.08
Item 26	Follow stop sign appropriately	0.03	0.08	-0.12	0.03	0.10	0.00	0.12	0.00	-0.02	-0.02	-0.14	0.04	0.01
Item 27	Follow yield situation appropriately	-0.11	0.25*	0.00	-0.02	-0.12	-0.09	0.00	0.10	0.02	-0.06	-0.15	-0.15	-0.11
Item 28	Slow down appropriately	-0.15	0.01	-0.22*	0.00	0.06	-0.04	0.10	-0.20*	-0.13	0.03	-0.04	-0.12	-0.08
Item 29	Start a car appropriately	-0.11	-0.13	-0.12	0.16	0.14	0.06	0.10	-0.13	-0.23*	-0.07	0.01	-0.03	0.05
Item 30	Reverse park appropriately	-0.20*	0.06	0.01	0.10	-0.03	0.10	-0.12	-0.15	-0.16	-0.08	0.01	-0.06	0.11
Item 31	Adjust driving speed appropriately	-0.11	0.05	-0.04	-0.08	-0.04	-0.05	0.04	-0.14	-0.07	0.12	-0.08	-0.15	-0.03
Item 32	Sudden brake appropriately	-0.03	0.16	-0.06	-0.04	-0.05	0.10	0.03	0.06	-0.16	0.01	-0.22*	0.08	0.04
Item 33	Drive pass the narrow street appropriately	-0.05	0.00	0.02	0.17	0.00	0.21*	-0.13	-0.17	-0.05	0.05	0.06	-0.02	-0.01
Item 34	Check rear view	-0.23*	-0.02	-0.02	0.30**	0.13	0.11	-0.17	-0.11	-0.38**	0.05	0.02	0.03	0.21*
Item 35	Check safety using head/body movement	-0.21*	0.22*	0.08	-0.05	-0.15	-0.22*	-0.22*	0.03	-0.02	0.07	-0.13	-0.07	-0.13
Item 36	Check safety when turning right	-0.10	0.04	-0.18	0.08	-0.03	-0.02	-0.09	-0.13	-0.12	0.08	-0.08	0.05	0.04
Item 37	Check safety when turning left	-0.10	-0.06	-0.13	0.07	0.09	0.04	-0.13	-0.21*	-0.13	-0.02	-0.13	0.12	0.16
Item 38	Check safety when changing lane/merging	-0.05	-0.07	0.00	0.06	-0.17	-0.11	-0.04	-0.02	-0.13	-0.02	0.00	-0.04	0.04
Item 39	Check safety when starting to drive	0.15	-0.06	-0.20*	0.02	-0.03	-0.04	0.02	-0.04	-0.10	0.05	-0.10	0.05	0.09
Item 40	Check safety in poor visibility	-0.01	0.00	-0.09	0.11	0.00	0.04	-0.04	-0.07	-0.01	0.07	-0.03	0.01	-0.07

Conversely, in terms of SOAD items, the greatest number of significant correlations with off-road tests was observed in items 34 and 35, both showing correlations with four tests, followed by item 13 with three tests. The highest correlation coefficient was found between J-SDSA dot error and item 36 (r = -0.37), followed by item 20 and dot time (r = -0.32) and item 34 and the KBDT (r = 0.30). All other significant correlations had correlation coefficients below 0.30.

## Discussion

The results of this study revealed generally weak correlations between SOAD and the various off-road assessments, particularly in evaluations of practical driving-related skills. This pattern supports the discriminant validity of SOAD by demonstrating its ability to evaluate unique driving capabilities that are not assessed by off-road tests. Discriminant validity refers to the degree to which a test or measurement distinguishes between the construct it aims to measure and other, potentially related constructs. It indicates that the assessment effectively captures distinct attributes, ensuring the measured skills are distinct from those in similar evaluations [12]. In the field of rehabilitation, discriminant validity is frequently established by calculating the correlations between assessments of distinct conceptual constructs [14,23]. In this study, we examined the discriminant validity by calculating correlations between off-road and on-road tests. The on-road test evaluates actual, multifaceted driving skills, while the off-road test assesses specific cognitive functions, each targeting fundamentally different constructs. Therefore, this methodology is appropriate, as it enables a clearer distinction between the specific capacities assessed by each test.

Correlated items suggest areas where SOAD aligns well with existing neuropsychological assessments (e.g., TMT and SDSA) [1,2], while uncorrelated items highlight the unique aspects of SOAD that are not assessed by conventional off-road tests. These findings underscore the complementary role of SOAD in driving evaluations and emphasize the importance of integrating both on-road and off-road assessments for a comprehensive evaluation of driving skills.

This finding aligns with prior studies, which suggest that off-road assessments primarily focus on cognitive domains such as visual processing and attention but fail to fully represent the complex skills required for safe driving [5]. For instance, some off-road tests measure isolated skills (e.g., visual scanning or attention switching), which may not encompass real-world driving demands, such as maneuvering in traffic, which requires continuous integration of sensory input, executive functioning, and motor response [23]. This fact suggests that items 34 and 35, which require advanced visual confirmation, had more significant correlations with off-road tests. Consequently, off-road tests may miss critical components of driving skills that SOAD captures, emphasizing the unique relevance of the on-road test within rehabilitation settings. The wide age range of participants (34-80 years) could be considered a potential source of variability in the study. However, age-related declines in both neuropsychological performance and driving skills are demonstrated, and these changes likely occur in parallel [24]. Therefore, the influence of age on the correlation between neuropsychological tests and driving skills may not significantly impact the validity of the discriminative analysis.

In Japan, where legal regulations restrict individuals with brain injuries from undergoing public on-road tests until they pass specific evaluations [17], the use of closed-course SOAD tests becomes especially significant. This regulation by the National Police Agency ensures that assessments are performed in a safer, controlled environment, where patients can demonstrate critical driving abilities, such as attentiveness and obstacle avoidance, without exposing themselves or others to risk on public roads. This closed-course setting aligns with local legal requirements and supports a thorough evaluation of practical driving competencies.

Furthermore, this scenario is not unique to Japan. Similar restrictions exist in several other countries, where legal policies or road infrastructure constraints limit on-road testing for individuals with cognitive impairments to closed courses [21]. In these cases, closed courses offer an effective alternative for assessing driving skills under realistic but safe conditions, though they differ from public road conditions. Despite being a simulation, closed-course testing allows for a comprehensive evaluation of essential driving skills. It provides a feasible and valid alternative to direct road testing in countries where legal frameworks limit public on-road access for specific populations.

The results of our study support the use of SOAD in such restricted settings, where it can assess distinct and essential driving skills not covered by off-road tests. The limited number of statistically significant correlations observed between SOAD and off-road assessments, combined with low correlation values, emphasizes the unique contributions of closed-course tests like SOAD in identifying practical driving skills essential for real-world scenarios.

While this study supports the discriminant validity of SOAD, its design is limited by the controlled setting of a closed course. To fully verify SOAD’s utility, future research could compare closed-course results with public-road driving assessments in countries where legal frameworks permit such comparisons. Additional studies that incorporate a public course would also contribute to a deeper understanding of the reliability and validity of SOAD.

## Conclusions

This study showed that there was a slight correlation between the off-road test and the SOAD in stroke individuals. This result indicates that the SOAD has minimal overlap with off-road cognitive assessments and highlights its discriminative validity. It demonstrates that the SOAD provides a valuable tool for assessing driving skills in a controlled environment. This study emphasizes its role as a reliable tool for evaluating the driving abilities of stroke patients, particularly in settings with legal constraints, such as Japan.
